# Enhancing the Electrical Conductivity of Electrospun PCL Fibers by Coating with Polydopamine and In Situ Gold Nanoparticles Doped on the Polydopamine Coating

**DOI:** 10.3390/polym17233192

**Published:** 2025-11-29

**Authors:** Taha Buğra Taşdelen, Özlem Eğri, Sinan Eğri

**Affiliations:** 1 Institute of Graduate Studies, Bioengineering Division, Tokat Gaziosmanpaşa University, 60250 Tokat, Türkiye; 2 Department of Mechanical Engineering, Faculty of Engineering and Architecture, Tokat Gaziosmanpaşa University, 60250 Tokat, Türkiye; 3 Department of Chemistry, Faculty of Science and Letters, Tokat Gaziosmanpaşa University, 60250 Tokat, Türkiye

**Keywords:** polydopamine, polycaprolactone, electrospinning, gold nanoparticles, electrical conductivity

## Abstract

Polycaprolactone (PCL) is a synthetic biodegradable polymer widely used in biomedical research due to its flexibility, safety for use in the body, and FDA approval for medical use. Nevertheless, its inherent hydrophobicity and restricted bioactivity limit its direct utilization in the field of biomaterials. Efforts to overcome these limitations include, but are not limited to, surface modifications, coating, and the use of copolymers of PCL with hydrophilic polymers. Polydopamine (PDA), the oxidative polymerization product of dopamine, a naturally occurring biomolecule in living organisms, is a flexible, bioinspired coating that makes surfaces more hydrophilic and facilitates cell attachment by incorporating numerous catechol and amine functional groups, making it suitable for biomaterial applications. PCL nanofibers were coated with PDA in three concentrations of dopamine solutions (0.2, 2, and 20 mg·mL^−1^). Then, gold nanoparticles (AuNPs) were deposited in situ using sodium borohydride reduction. Morphological, physicochemical, and electrical properties of both PDA-coated and AuNP-loaded PCL fibers were comparatively investigated. The PDA coating made the surface significantly more hydrophilic compared to PCL-only surfaces, and AuNP-loaded fibers exhibited an extremely hydrophilic character. The primary concern of this article, electrical conductivity, was found to increase by up to a hundredfold with PDA coating and by a thousandfold with loading of AuNPs. PDA coating or loading AuNPs onto PDA-coated electrospun PCL fibers can provide a wide range of applications in the field of biomaterials.

## 1. Introduction

Bioelectrical signals are vital for many physiological functions in the body. Electroactive scaffolds can naturally exhibit bioactivity by interacting with the body’s own electric fields, even in the absence of external electrical stimulation (ES). Nonetheless, the combined use of external ES and these conductive scaffolds has been shown to improve cell adhesion, proliferation, and lineage specification significantly. This combined method has been demonstrated to be effective in various areas of tissue engineering, including bone, skin, neural, skeletal muscle, and cardiac tissue [1]. Polymers of dopamine, which possess adhesive properties similar to those of mussel adhesives, offer a unique and effective means of modifying the surfaces of biomaterials. At present, polydopamine (PDA) has been successfully applied to the surfaces of various materials, including metals, ceramics, polymers, and even living cells. The most important aspect is that the PDA coating serves as a flexible “platform” (or intermediate layer) for the secondary immobilization of other functional molecules [2]. PDA, the ultimate oxidation product of dopamine and other catecholamines, gained considerable interest as a multipurpose coating capable of uniformly covering the surfaces of nearly all materials with a tunable thickness ranging from a few nanometers to over 100 nanometers [3]. Due to this unique ability, it has attracted the attention of researchers to modulate the surfaces by coating PDA [4].

Polycaprolactone (PCL) is a synthetic, biodegradable polymer approved by the FDA for use in various biomedical applications. Polycaprolactone (PCL) is distinguished by its remarkable mechanical qualities, capacity to create copolymers with other polymers, and biodegradability [5]. PCL exhibits a melting point of 60 °C and a glass transition temperature of −60 °C, which is essential to its favorable polyester configuration. Due to its biodegradability, low melting point, and compatibility with other polymers, PCL has been extensively researched for its possible applications in biomedical fields [6].

Electrospinning is a simple, cost-effective, adaptable, and innovative technology that attracts significant interest in academia and biomedical applications [7]. The distinct characteristics of electrospun fiber scaffolds include a high surface area-to-volume ratio, superior porosity, consistent fiber structure, compositional diversity, flexibility, and the ability to be functionalized with bioactive molecules. PCL’s versatility enables it to be efficiently processed into nanofiber structures using the electrospinning technique [8].

Nanofiber matrices functionalized at the surface and exhibiting specific surface properties are of significant interest, potentially expanding their applications. These surface properties can influence wettability, electrical conductivity, optical properties, and biocompatibility. Various methods, including physical, chemical, and biological approaches, have been employed to design nanofibers with modified surface properties [9]. By applying a PDA coating to the scaffold, the researchers significantly improved cell adhesion, thereby enabling these adhesive properties to be utilized in various biomedical applications, particularly in the field of medicine [10]. PDA has been effectively employed to enhance adhesion, owing to its rich array of functional groups that enable the covalent binding of biomolecules and the anchoring of reactive species and ions [11]. PDA, a polymer not soluble in water, is highly cross-linked, resulting from the induced oxidation of dopamine monomers in alkaline environments [12,13]. Consequently, PDA coatings that demonstrate excellent adhesion, secondary reactivity, hydrophilicity, and biocompatibility traits are utilized in biomedical applications, resulting in a favorable outlook for the future [14,15].

The continuous advancement in nanomedicine has led to a growing interest in gold nanoparticles, which are known for their ease of synthesis and functionalization. Gold nanoparticles (AuNPs), which have a high surface area-to-volume ratio, are biocompatible, can be used in medical imaging, are non-toxic, can enter cells, are highly reactive, and possess unique optical and electronic properties. They have made a significant impact on nanomedicine over the last few years. AuNPs have been utilized in various biomedical applications, including localized hyperthermia to induce apoptosis in cancer cells, medical imaging, drug delivery, and tissue engineering for skeletal muscle, cardiac tissue, and bone tissue [16,17]. AuNPs are excellent candidates for enhancing conductivity and promoting electrical coupling among cells because of their exceptional electrical conductivity, chemical stability, and biocompatibility [18]. AuNPs exhibit potential antioxidant and/or antimicrobial activities, which enhance their other beneficial attributes. By acting as scavengers of free radicals, these nanoparticles can reduce oxidative degradation in polymers [19]. By forming hybrid nanocomposites of AuNPs with polymers that provide additional functions, responsiveness, and improved biocompatibility, the capabilities of AuNPs can be enhanced [20].

In this study, the aim was to enhance the surface properties, especially the electrical conductivity, of the PCL nanofibrous matrices produced with the electrospinning technique. To the best of our knowledge, the electrical properties of the PDA-coated electrospun matrices have not been reported before. The electrical properties of PDA-coated PCL nanofibers have been investigated in this study to provide an alternative insight into their potential biomedical applications. For this purpose, three main experimental groups—PCL fibers only, PCL fibers with surface coatings of PDA (coated in 0.2 mg·mL^−1^, 2 mg·mL^−1^, and 20 mg·mL^−1^ solutions), and PDA-coated fibers with gold nanoparticles incorporated onto their surfaces—were investigated.

## 2. Materials and Methods

Electrospun PCL fibers were produced from Sigma Aldrich (M_n_ = 80,000 Da, St. Louis, MO, USA) PCL pellets. PDA was coated on the surface of PCL fibers using dopamine hydrochloride from Alfa Aesar (99%), Kandel, Germany; HAuCl_4_ (gold(III) chloride, 99.99% trace metals basis), which is used as a precursor for gold nanoparticles (Sigma-Aldrich, St. Louis, MO, USA); and sodium borohydride (NaBH_4_), which is used for in situ gold nanoparticle reduction, sourced from Carlo Erba (Milano, Italy). Seven groups were abbreviated as PCL, 0.2PDA, 2PDA, 20PDA, 0.2PDA-Au, 2PDA-Au, and 20PDA-Au.

### 2.1. Electrospun PCL Fiber Production

The polymer solution was prepared using 1.8 g of PCL (80 kDa), 10 mL of chloroform, and 2 mL of ethanol, resulting in a concentration of 15% w·v^−1^. It was then stirred for 24 h in a magnetic stirrer before being spun using electrospinning equipment (Gamma High Voltage Res. Inc., Ormond Beach, FL, USA). After preparation, a 10 mL syringe with a 21-gauge stainless steel needle was filled with the homogeneous polymer solution. In order to control the flow rate of the polymer solution during the electrospinning procedure, the syringe was subsequently connected to a programmable syringe pump. A standard electrospinning apparatus consisting of a grounded rotating collector, a syringe pump, and a high-voltage power source was used to carry out the electrospinning process. A flow rate of 2 mL·h^−1^, a voltage of 14 kV, and a distance of 10 cm between the tip and the collector (9 cm diameter, 150 RPM rotation rate) were determined to be the ideal electrospinning parameters.

### 2.2. PDA Coating of PCL Fiber Surfaces

A Tris buffer solution with a pH of 8.5 was first prepared to coat the surfaces of electrospun PCL fibers with PDA. The buffer was prepared by dissolving 10 mM Tris(hydroxymethyl)aminomethane in deionized water, followed by pH adjustment to 8.5 with hydrochloric acid. Subsequently, the PCL fibers produced by electrospinning were obtained by discarding the parts outside the central 2.5 × 10 cm dimensions. These fibers were washed with distilled water and dried. Solutions containing dopamine-HCl were prepared at concentrations of 0.2 mg·mL^−1^ (0.2PDA), 2 mg·mL^−1^ (2PDA), and 20 mg·mL^−1^ (20PDA) in a Tris-Buffer solution (pH 8.5), and PCL fibers were immersed in these solutions and left to stand for 16 h. After 16 h, the PCL fibers were removed from the solutions, washed with distilled water, and dried under atmospheric conditions.

### 2.3. Loading AUNPs onto the Surfaces of PDA-Coated PCL Fibers

HAuCl_4_ was used as a gold precursor to deposit gold onto the surface of PDA-coated electrospun fibers. Gold nanoparticles were deposited onto the surface of PDA-coated PCL fibers using an in situ method. HAuCl_4_ solution with a pH of 4 and a concentration of 0.2 mM was prepared to deposit gold nanoparticles onto the surface of PCL fibers coated with PDA at concentrations of 0.2 mg·mL^−1^ (0.2PDA-Au), 2 mg·mL^−1^ (2PDA-Au), and 20 mg·mL^−1^ (20PDA-Au). The fibers were brought to the specified size in the PDA coating, washed with pure water, and then dried. The fibers were kept in a solution containing 0.2 mm HAuCl_4_ for 1 h after being dried, and the gold ions were connected to the functional groups in PDA. After one hour, the fibers were immersed in a fresh, ice-cold 2 mM NaBH_4_ solution and left for five minutes. After this period, the fibers were washed with distilled water and left to dry.

### 2.4. Characterization of Surface Functional Groups

In order to characterize the changes in the chemical structure of the fibers produced in all experimental groups, the FTIR-ATR (Fourier Transform Infrared Spectroscopy–Attenuated Total Reflection, Jasco, FT-IR/4700, Tokyo, Japan) spectra of the fiber matrices were collected between 4000 and 400 cm^−1^ wavelengths separately and examined. The fibers were placed on a diamond disk and fixed to the disk surface under pressure. The absorption of the IR beam sent to the surface by the material, as well as the IR intensity reflected from the crystal, was recorded.

### 2.5. Scanning Electron Microscope (SEM) Analyses

Circular sections of 6 mm diameter were taken from the matrices, and their surfaces were coated with Au under a vacuum atmosphere (Quorum Q150RES, Birmingham, UK) for 10 min to render the sections conductive. Subsequently, SEM (Tescan Mira3 XMU, Brno, Czech Republic) images were obtained at different magnifications in order to analyze their surface morphologies.

### 2.6. Density and Porosity

Five square sections, prepared with 1 × 1 cm dimensions, were taken from the fiber matrices, and the density (ρ) and average specific volume (cm^3^·g^−1^) of these fiber matrices were calculated using the weight (g) and volume (cm^3^) values. Then, the % porosity values were calculated using Equation (1) [21].

Porosity (%) = [(V_m_ − V_0_)/V_m_] × 100 (1)

where V_0_ represents the specific volume (cm^3^·g^−1^) for the pure polymeric film and V_m_ represents the specific volume (cm^3^·g^−1^) values for the electrospun polymeric patches.

### 2.7. Water Contact Angle

The contact angle test measurements of the samples were performed using a water contact angle device (Attension, Biolin Scientific AB, Gothenburg, Sweden) in accordance with the ASTM D7334-2013 standard [22]. For the determination of contact angles, a 4 μL droplet of deionized water was dropped onto the surface of each sample, and a total of 21 samples out of 7 groups were tested. Images of the water droplets on the surface were captured using a camera, and the contact angle values were determined for each sample by calculating the average of the measured values. The hydrophilic/hydrophobic properties of each sample were then evaluated.

### 2.8. Swelling Tests

The swelling tests were performed using electrospun matrices with circular sections of 6 mm in diameter by weighing the dry and wet samples, immersed in PBS for intervals of 3, 6, 9, 12, 15, 30, 60, 90, and 120 min, and subsequently removing the excess water from the surface. The swelling ratio was calculated using Equation (2) [23].

Swelling Ratio (%) = [(W_i_ − W_0_)/W_0_] × 100 (2)

where W_0_ and W_t_ represent the dry and wet weights of the samples, respectively.

### 2.9. Mechanical Strength

A tensile testing device (Autograph AGS-X, Shimadzu, Kyoto, Japan) was used in accordance with EN ISO 13934-1:2013 [24] to determine the mechanical strength of the produced electrospun matrices. Samples cut in 0.5 cm × 10 cm dimensions were fixed between the sample holders, and the measurements were conducted at room temperature with a constant tensile speed of 50 mm·min^−1^. Upon testing, the tensile stress (stress) versus strain values (strain) of the samples were recorded. The ultimate tensile strength (UTS), fracture strain, and elastic modulus values were determined for all groups. Five samples were tested for each group, and the average and standard deviations were calculated. A representative photo is presented in Figure S1, which shows the test specimens before and during tensile testing.

### 2.10. Electrical Conductivity

The four-probe conductivity test was performed on each sample group in triplicate measurements. The sheet resistance values were then determined, and their conductivities were evaluated using Equation (3) [25].

σ = [ln2/(πt)] × (I/V)(3)

where σ represents electrical conductivity, I represents applied current, π represents the Pi constant, t represents film thickness, and ν represents measured voltage.

### 2.11. Statistical Analyses

The data were statistically analyzed within the context of this study using the one-way ANOVA variance analysis method (IBM SPSS Statistics 27, IBM Corp., Armonk, NY, USA), followed by post hoc multiple tests (Tukey, Bonferroni). 

## 3. Results

### 3.1. Characterization of Surface Functional Groups

**Figure 1:**
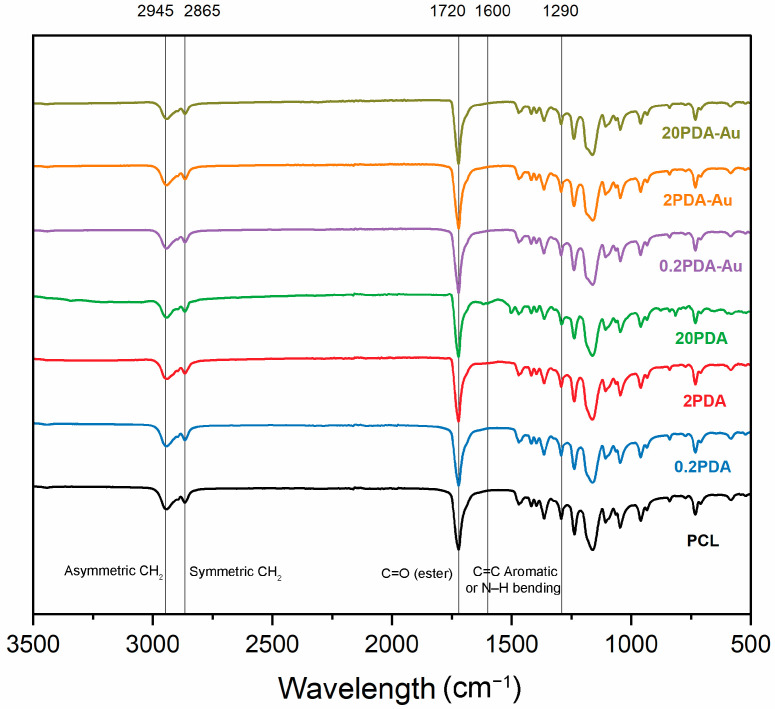
FTIR spectra of all experimental groups.

FTIR spectra of PCL and PCL samples coated with PDA on the surface by immersion for 16 h at concentrations of 0.2 mg·mL^−1^, 2 mg·mL^−1^, and 20 mg·mL^−1^ dopamine hydrochloride solution, and the in situ formed AuNP-loaded forms are shown in Figure 1.

For PCL fibers, asymmetric and symmetric -–CH_2_–- stretching bands at 2945 cm^−1^ and 2865 cm^−1^ [26], an ester carbonyl (C = O) stretching band at 1720 cm^−1^ [27], and characteristic peaks of C–O–C stretching bands at 1290 cm^−1^, 1238 cm^−1^, and 1170 cm^−1^ [28] were observed at the so-called wavenumbers. These peaks confirm the chemical structure of PCL for all samples in the experimental groups.

For the fibers coated with PDA, several new peaks appeared, and the intensities of the existing bands changed as the concentration increased. In particular, a broader band corresponding to O–H and N–H groups was observed between the 3200–3500 cm^−1^ range, and this band increased in intensity alongside the density of the PDA coating. Moreover, a band corresponding to aromatic C = C and/or N–H bending vibrations was detected in the ~1590–1610 cm^−1^ region [11]. Notably, the peak that becomes visible around 1500 cm^−1^ for the 20PDA group corresponds to the skeletal vibrations of the aromatic rings of PDA and/or secondary amine groups, reflecting the structural characteristics of PDA, and this does not exist for PCL. This peak indicates the formation of a dense and uniform PDA layer on the surface. These bands are not distinguishable within the whole spectra, as the amount of PDA coated on the PCL surface is tiny compared to the gigantic PCL structure so that the change in intensity cannot easily be noted for 0.2PDA and 2PDA. Similarly, at higher concentrations, bands in the ~1250–1280 cm^−1^ region, corresponding to C–N stretching, became more noticeable. These results demonstrate that PDA adheres well to the fiber surface and that increasing concentration enhances coating process efficiency. The bands are notably weak for 0.2PDA; however, they are significantly enhanced for 2PDA and especially for 20PDA. Moreover, bands related to C–N stretching in the ~1250–1280 cm^−1^ range are more distinctly observed for samples coated in higher concentrations of dopamine solutions. These results imply that PDA adheres effectively to the fiber surface, with the coating process becoming more effective as the concentration increases. There was no observed shift or decrease in intensity of the characteristic peak at the 1722 cm^−1^ ester carbonyl band of PCL in the FTIR spectra. This supports the conclusion that the PDA coating does not alter the fundamental structure of the fibers but instead provides surface modification.

A wide stretching band for OH (phenolic) and NH (amine) is visible in this region (3200–3500 cm^−1^) for PCL and PDA-coated samples without gold nanoparticles. On the other hand, these bands are much more noticeable and have a higher peak intensity in PDA-coated samples loaded with gold. This broadening is attributed to the interaction between gold nanoparticles and the phenolic –OH groups of PDA, as well as the coordination of the amine groups with the gold nanoparticle surface. The interaction of gold with these functional groups broadens and enhances the peaks in these regions. Such interactions suggest that PDA establishes chemical bonds with the gold surface, reinforcing and stabilizing the structure.

In the PDA-PCL sample that includes gold nanoparticles, a reduction in intensity and a loss of sharpness in the CH_2_ peak were noted (2945–2865 cm^−1^). This alteration suggests that gold nanoparticles interact with the aliphatic C–H bonds on the PCL surface, causing structural alterations around this group and potentially producing a weaker or more flexible bond structure inside the aliphatic group.

In the FTIR spectrum of pure PCL, a distinct and pronounced peak corresponding to the C = O (carbonyl) group is observed (1720 cm^−1^). This peak is prominently visible in the PDA-PCL sample, which does not contain gold nanoparticles. Conversely, in PDA-PCL samples that include gold nanoparticles, a minor reduction in intensity and a positional shift in the C = O peaks have been noted. This alteration may be attributed to the interaction between gold nanoparticles and the carbonyl group of PCL. The presence of gold can modify the electron density surrounding this group, resulting in a weakened bond structure of the carbonyl group and a subsequent decrease in its intensity in the FTIR spectrum.

PDA’s C = C and NH_2_ bending bands (nearly 1600 cm^−1^) are prominently observed, especially in PDA-coated PCL samples that lack gold nanoparticles. On the other hand, the peaks in this area become sharper and more intense in PDA-PCL samples that contain gold. Moreover, it is hypothesized that the NH_2_ groups interact with the gold nanoparticles, resulting in increased reflection in this area.

FTIR spectra effectively demonstrate the chemical interactions formed by gold nanoparticles on PDA-coated PCL. The usual PCL and PDA peaks detected in gold-free PDA-PCL samples exhibit clear changes with the introduction of gold nanoparticles. Notably, there is broadening in the OH, NH, and aromatic C = C groups, a decline in the intensity of the C = O group, and a significant rise in the peaks of the aromatic structure. These results suggest that gold nanoparticles modify the surface properties of PDA-coated PCL fibers and form chemical bonds, and that these interactions can alter the physical, chemical, and biological properties of the fibers.

### 3.2. SEM Analysis

**Figure 2:**
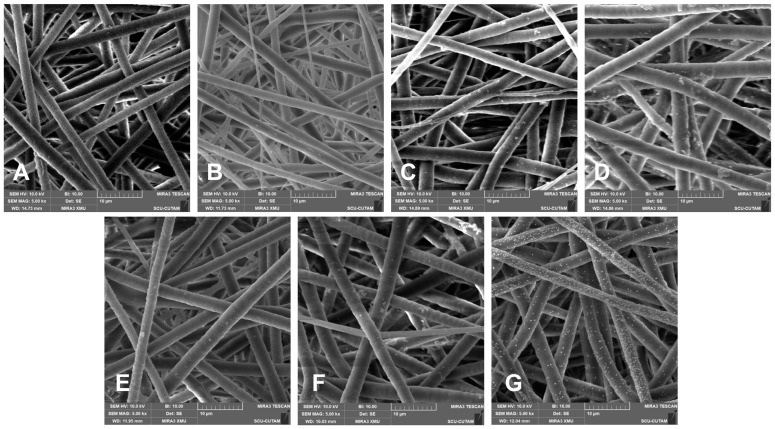
Representative SEM images of electrospun fibers: (**A)** PCL; (**B**) 0.2PDA; (**C**) 2PDA; (**D**) 20PDA; (**E**) 0.2PDA-Au; (**F**) 2PDA-Au; and (**G**) 20PDA-Au.

Scanning electron microscope (SEM) images were obtained and evaluated for samples belonging to the seven experimental groups and presented in Figure 2. All images represented in this figure demonstrate the morphological changes attributed to the PDA coating and in situ loading of AuNPs.

The PCL fibers (A) display a consistent morphology, a uniform distribution, and a relatively thinner diameter of 2.72 ± 0.50 µm. After subjecting a low concentration of PDA coating (0.2PDA), a slight increase in roughness, together with tiny clusters unevenly distributed, was noted on the fiber surface, suggesting that PDA adhered to the surface (Figure 2B). At this point, there was no significant increase in fiber diameter. When the PDA coating solution concentration was raised tenfold (Figure 2C), the fibers exhibited a thicker morphology of 3.20 ± 0.12 µm, and the surface roughness became more evident with an increasing number of PDA clusters. At the highest PDA coating solution concentration, increased to a hundredfold (Figure 2D), the fiber diameters increased considerably to 3.60 ± 0.14 µm, resulting in more compact, thicker, and coated structures due to the dense coating layer, along with an increase in the number and size of the clusters. These findings clearly indicate that an increase in PDA concentration has a direct impact on fiber diameter and surface morphology, leading to the formation of coarser, thicker structures in relation to coating density, together with the increasing number of clusters, which are concluded to be the freely crosslinked PDA particulates of excess dopamine in the coating solution that are bonded to the PDA-coated fibers by secondary interactions that cannot be removed from the surface by repetitive washing.

The SEM images of AuNP-loaded PDA-coated fibers (Figure 2E–G) clearly indicate that as the thickness of the PDA coating increased, the adhesion of AuNPs onto the fiber surface and the density of the loading also increased. This behavior can be attributed to the chemical structure of PDA, where the catechol and amine groups in the PDA structure can form binding sites on the surface by complexing metal ions. Additionally, PDA can provide nucleation centers, as it tends to reduce Au^3+^ ions locally. Consequently, as the PDA density increases, more chemical binding sites and nucleation areas appear on the fiber surface. Furthermore, when the in situ reduction method is added to this arrangement, a higher number and more homogeneous distribution of AuNP bonding can occur. Additionally, the PDA layer facilitates the deposition of metal ions on the fiber surface and supports the continuity of the coating by altering the surface wettability and surface energy. It is evident that PDA particulate clusters on the PDA-coated fibers were noticeably decreased for AuNP-loaded samples. This behavior was attributed to the breaking of the linkage between the PDA-coated surface and the PDA particulate clusters due to the treatments at acidic and alkaline pH values during AuNP loading. The SEM images of the PDA-coated fibers after Au deposition were examined, and the particle size was calculated from multiple regions. The Au particles exhibit an average size of approximately 74±16 nm, with a size range of 44–99 nm.

### 3.3. Density and Porosity

**Figure 3:**
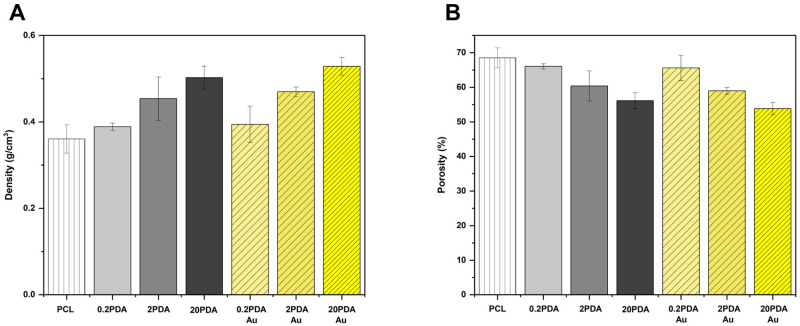
Density (**A**) and porosity (**B**) of the electrospun fibers.

Electrospun fibers were subjected to density and porosity calculations using Equation (1), and the results were plotted on the graph presented in Figure 3.

As observed in the SEM images, the fiber diameters of electrospun PCL fibers increase with the concentration of dopamine hydrochloride solution used in the dip-coating process. A similar correlation was also observed in the density calculations. This behavior indicates that as the coating concentration increases, the density of the electrospun fibers also increases. Additionally, although to a lesser extent, the density can also be affected by the gold nanoparticle loading process, but the concentration of AuNPs does not make a significant change. Contrary to density, an increase in dopamine hydrochloride solution concentration results in a decrease in porosity, which correlates with an increase in density, depending on the fiber thickness. The porosity is maximum for PCL fibers, diminishing in the sample groups including 20PDA and 20PDA-Au. This scenario may be attributed to the increase in fiber thickness. The loading procedure for AuNPs slightly reduces porosity.

### 3.4. Water Contact Angle Measurements

Water contact angle measurements were performed on sample groups, and the average contact angles, along with their corresponding standard deviations, were determined in triplicate for each group (representative photos for each group are presented in Figure S2). The results are summarized in Table 1.

A correlation is observed between the amount of PDA coated on the surface and the decrease in contact angle, as indicated by the contact angle data. PCL fiber exhibits a hydrophobic character, with an average contact angle of 99.19°. The 0.2PDA group showed an average contact angle similar to that of the PCL-only group, displaying similar characteristics. This indicates that PDA coating at a lower concentration has an insignificant effect on the contact angle. However, the contact angles significantly decreased to 62.62° and 17.77°, respectively, when the coating solution concentration was increased tenfold and hundredfold, suggesting that the amount of PDA significantly increased the hydrophilicity. Depending on the PDA concentration, the wettability was further impacted by the addition of AuNPs. The contact angle dropped to 28.77° for 0.2PDA-Au and 19.35° for 2PDA-Au. When applying water droplets to 20PDA-Au, the contact angle was observable during initial contact; however, it became unmeasurable once the droplet had suddenly disappeared entirely. The relevant test video is provided as a Supplementary Material (Video S1). These results demonstrate that the hydrophilicity of PCL nanofibers is enhanced by both increased PDA coating thickness and the inclusion of Au nanoparticles, with the most significant effect being obtained at the highest PDA content combined with gold.

### 3.5. Swelling Behavior

The PCL group has the least swelling capacity, and the rate of swelling was stabilized after 60 minutes. This observation is consistent with the hydrophobic properties of PCL, as well as the capillary effect resulting from the interconnected pores between fibers. The 0.2PDA group exhibited a significantly higher swelling rate compared to the PCL group. This improvement suggests that a low concentration of PDA coating results in a hydrophilic surface effect. The 2PDA group had a swelling capacity that had increased more noticeably due to the PDA coating. The increased amount of amine and catechol groups on the fiber surface likely enhanced its ability to retain water. The 20PDA group exhibited excellent swelling capacity. The fact that the swelling was 1500% suggests that the thicker PDA coating produced an extremely hydrophilic environment, which helped the fibers retain this amount of water. 

**Figure 4:**
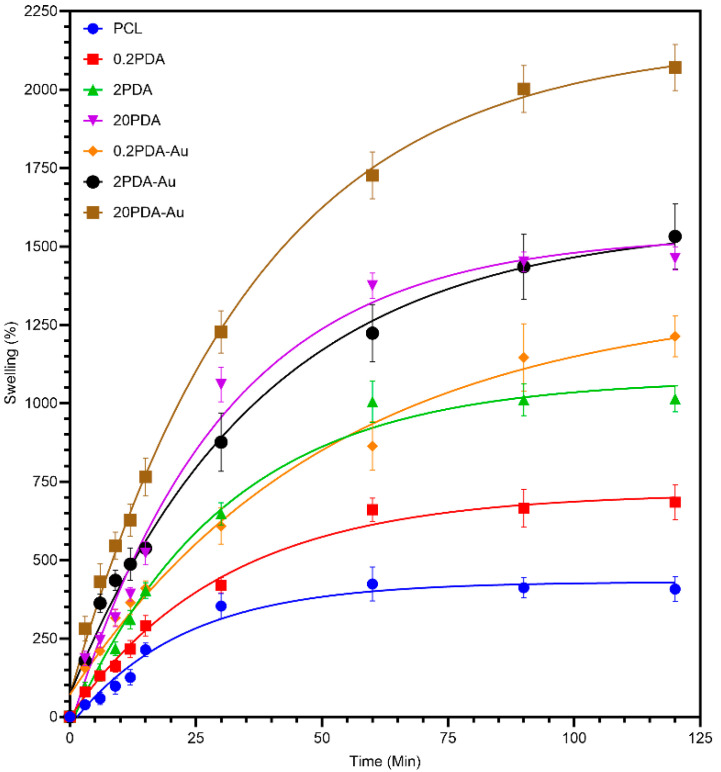
Swelling behavior of electrospun PCL fibers.

According to Figure 4 and the results previously presented, the loading efficiency of AuNPs increased with the increase in PDA coating thickness, which further improved the surface hydrophilicity. The water retention capacity more noticeably increased compared to those coated with PDA only. This phenomenon can be explained by the higher PDA concentration on the surface, which results in an increase in surface energy due to the gold nanoparticles, leading to increased surface hydrophilicity [29]. Alternatively, it can also be said that tertiary and secondary amines were induced to higher amines by acid/alkaline treatment while AuNP loading, which may result in enhanced hydrophilicity [30].

### 3.6. Mechanical Strength

The tensile test was conducted on a universal testing machine in accordance with the relevant standard. The ultimate tensile strength (UTS), fracture strain, and modulus of elasticity values for all groups are collectively presented in Table 2 (representative load-deformation graphs obtained from the Instron device for each group are presented in Figure S3). According to the results summarized in Table 2, the tensile strength of electrospun PCL fibers was determined to be 7.66 ± 1.53 MPa, elongation at break as 4.83 ± 0.44, and the elastic modulus as 43.93 ± 3.24 MPa, which are found to be consistent with previous works [31]. After PDA coating, concentration-dependent and -proportional changes in mechanical properties were observed. For PDA coating at low concentrations (0.2PDA) and its AuNP-loaded form (0.2PDA-Au), a slight change was observed in all three properties, which can be attributed to PDA coating and AuNP loading; however, these values are very close to those of PCL. As the PDA coating thickness increases, it results in an increase in UTS and elastic modulus and a decrease in fracture strain. The increase in UTS and elastic modulus can be attributed to the increasing amount of cross-linking in PDA and secondary interactions between the functional groups of PDA, resulting in a decrease in ductility, specifically, the fracture strain. By the addition of AuNPs to the PDA-coated fibers, UTS decreased compared to PDA-coated fibers under the same conditions. Fracture strain and elastic modulus exhibited a correlated behavior (opposite to each other) since an increase in AuNP density on the PDA-coated fiber surface resulted in the lowest fracture strain (4.21 ± 0.10 mm·mm^−1^) and the highest modulus of elasticity (49.71 ± 0.21 MPa). This can be attributed to the AuNPs’ growing interactions with surrounding functional groups by PDA as the density of AuNPs on the fiber surface increased.

Based on the statistical analyses performed on the mechanical test data, the significance of the difference between the maximum tensile stress values was determined to be *p* = 0.477. Since this value is greater than 0.05, it can be concluded that there is no significant difference between the UTS values of the groups (the relevant one-way ANOVA test result is provided in Table S1). Similarly, no significant change was observed for fracture strain values between groups (*p* = 0.088) (Table S2). When statistical analysis was applied between groups for the modulus of elasticity, a significant difference was observed between at least two groups (*p* = 0.019) (Table S3). Accordingly, post hoc multiple analyses were performed (Tukey, Bonferroni). According to Tukey HSD post hoc multiple analysis tests, a significant difference was observed between the PCL and 20PDA-Au groups (*p* = 0.038); however, no other significant differences were determined between the different groups. However, according to Bonferroni post hoc multiple analysis tests, the significance value between these two groups was determined to be 0.067, indicating no significant difference between them. 

Overall, PDA coating increases the stiffness and strength of PCL fibers in a concentration-dependent manner. At the same time, Au nanoparticles modulate mechanical performance, improving stiffness at high concentrations and ductility at low concentrations. However, the collective results showed no significant changes in mechanical properties at the macro scale.

### 3.7. Electrical Conductivity

A four-probe conductivity test was performed on the electrospun fibers, and the sheet resistance values were determined, enabling the evaluation of their conductivity. The calculations for electrical conductivity were performed using Equation (3), and the related results are summarized in Table 3.

The results presented in the table clearly indicate that PCL-only electrospun fibers exhibit very low surface conductivity, falling within the category of insulators. Conductivity increases correspondingly as the PDA concentration on the surface coating increases. The formation of a thicker, more uniform, rough surface that promotes less impeded electron mobility may result from increased PDA concentration, which also increases the number of active functional groups. PDA’s aromatic structure could result in π-π interactions that promote electron delocalization.

Samples coated with PDA and loaded with AuNPs showed elevated conductivity values. This can be attributed to the binding of AuNPs to catechol and amine groups within the PDA structure, which can act as a conductive bridge for electrons, thereby significantly increasing conductivity (*p* = 0.001). The surface conductivity of AuNP-loaded materials increases proportionally to the increase in PDA thickness. These two findings are supported by SEM images, which show that the amount of gold nanoparticle attachment increases as the PDA thickness develops. 

## 4. Discussion

In our study, we investigated the changes in the chemical, electrical conductivity, and mechanical properties of electrospun PCL fibers by coating their surfaces with PDA at different concentrations and loading AuNPs onto the PDA-coated surfaces. When examining the FTIR results, the FTIR peaks of PCL fibers yielded similar results to those reported previously [32,33]. The broadening of the N–-H and O–-H bands and the C = C and/or N–-H bending peaks that became apparent around 1600 cm^−1^ as a result of coating the surfaces of PCL fibers with PDA indicated that the PDA coating was successful, and the results were consistent with the literature [11]. Furthermore, the broadening of the O–H/N–H band and changes in the intensity of some bands after in situ AuNPs’ reduction on the PDA coating support the possibility of binding/reducing interactions between the catechol/amine groups of PDA and the Au surface. It has been reported that PDA complexes reduce metal ions and bind AuNPs to the surface of AuNPs [34]. In summary, FTIR analysis reveals that AuNP loading has a significant impact on the surface chemistry through interactions with PDA and the metal and that PDA coats PCL in a concentration-dependent manner. These findings are consistent with previous reports and offer strong support for the PCL/PDA/Au triple combinations [35].

The morphological analyses, conducted using SEM image analysis, showed that fiber diameter and surface roughness increased in tandem with PDA concentration. At a coating concentration using 20 mg·mL^−1^ dopamine solution, the maximum fiber diameter was observed. PDA optimization studies involving PCL revealed similar thickening and topological changes [11]. The quantity of AuNPs, which adhere strongly and uniformly in direct correlation to the PDA coating concentration, aligns with PDA’s capacity to complex and reduce metal ions while offering numerous binding sites. This mechanism is traditionally recognized in the in situ reduction in AuNPs on PDA coatings [34,35]. Considering all these results, PDA coating can be regarded as an effective tool for controlling the morphology and coating efficiency of AuNPs. Therefore, PDA concentration can be evaluated as a practical and direct parameter for adjusting the desired Au coating density. In conclusion, SEM images qualitatively support the notion that Au adhesion clearly increases as the PDA concentration in the coating increases.

The hydrophobic behavior of pure PCL is a well-known phenomenon, and the measured value measures this hydrophobic behavior [36]. The decrease in contact angle and transition to hydrophilic limits with increasing PDA coating concentration is consistent with the literature. The literature also reports the creation of super-hydrophilic structures using different treatments in addition to the PDA coating process [37,38]. For the 20PDA-Au group, complete wetting was observed at the moment of contact between the water droplet and the surface, making the contact angle unmeasurable. This behavior can be attributed to the formation of a denser and hydrophilic active surface due to PDA’s metal-binding/nucleation ability, together with the capillary effect resulting from interconnected pores along the fibers. Swelling test results have also supported this conclusion, where the water retention capacity increased with increasing PDA coating thickness and AuNP loading. The sharp increase in water retention capacity as the PDA coating thickness and AuNP binding amount increased can be attributed to the rise in surface energy and water stabilization through hydrogen bonding by catechol/amine functions. Similar behaviors have been reported in PDA-modified polymer/membrane systems [39].

The effect of PDA coating on the mechanical properties of PCL fibers appears variable in the literature and depends on the structure created. In some studies, PDA coating can increase modulus/strength (due to inter-fiber adhesion/bonding), while softening/decreasing has been reported in other cases. According to the data obtained in our study, the PDA coating resulted in a limited but significant increase in modulus and UTS. In contrast, a slight decrease in elongation at break (increased brittleness) was observed. The resulting condition is consistent with systems where inter-fiber friction and coating hardness have increased [40]. When AuNPs were added, the increase in elongation at break at low PDA levels (possible “plasticizer/carrier distribution” effect) and the slight decrease in elongation at maximum modulus at high PDA + Au levels can be explained by the limitation of nanoparticle mobility within a rigid network. Similar balancing effects have been reported in nanoparticle-loaded PCL fiber mats [41].

It is well known that pure PCL fibers are electrically insulating/have extremely low conductivity. A significant increase in electrical conductivity was expected with PDA coating on the surfaces of PCL fibers. Even greater increases in electrical conductivity were expected due to the loading of AuNPs. In this study, a significant increase in electrical conductivity was observed depending on the PDA coating concentration (ranging from 10^−7^ to 10^−6^ S·cm^−1^ levels), and an exit to the 10^−6^–10^−5^ S·cm^−1^ band was observed with AuNP loading on the PDA coating. This pattern aligns with studies indicating that PDA facilitates carrier mobility through its aromatic π–π networks and N-containing groups, and that AuNPs act as conductive bridges [42,43]. The mechanism that exhibits an increase in electrical conductivity by the PCL/PDA/AuNP system can be attributed to a dual-mode charge transport process involving “hopping conduction” and “interfacial charge transfer”. Pure PDA is an organic semiconductor in which charges propagate by hopping from one molecular ring to another [44]. By doping AuNPs onto the PDA surface, this process is eased by the action of AuNPs as stepping bridges that reduce the hopping distance for charge transfer. As the second mode, when a metal (Au) and a semiconductor (PDA) with different Fermi levels come into contact, charge injection occurs from the metal to the polymer (or vice versa), resulting in the formation of a charge accumulation layer at the interface between the AuNPs and the PDA. 

In addition, although no study on the electrical conductivity change in structures containing the PCL/PDA/AuNP triple effect or any of these two has been identified in the literature, it has been reported that an increase in electrical conductivity was observed by doping gold nanoparticles into the PCL/chitosan nanofibrous structure [16]. Additionally, our recent findings indicate that the presence of metal ions/partial dopants loaded onto the PDA coating can improve electrical properties. These findings provide an additional framework explaining the conductivity gains observed in coatings with high PDA concentration in combination with AuNPs [45].

## 5. Conclusions

This study investigates the individual effects of PDA surface coating on PCL fibers at concentrations of 0.2, 2, and 20 mg·mL^−1^ dopamine solutions, followed by loading with AuNPs in situ, using 0.2 mM HAuCl_4_ solution, and (i) the individual effects of PDA concentration on FTIR analysis, wettability/swelling, morphology, mechanical properties, and surface electrical conductivity, and (ii) the additional/synergistic effects of in situ AuNP loading under the same experimental conditions, as the enhancement in electrical conductivity was the primary concern. 

Chemical characterization using FTIR analysis confirmed the successful coating of PDA on the PCL fibers, with changes in the specific bands attributed to the N–H, OH, and C=C groups of PDA. Morphological evaluations also confirmed the successful PDA coating, as evidenced by the change in diameter of the PCL fibers, which was supported by an increasing density and decreasing porosity. The hydrophilicity exhibited a significant increase with increasing coating solution concentration, which was also enhanced by the in situ AuNP loading process. The 20PDA-Au group exhibited almost super-hydrophilic behavior, which prevented stable contact angle measurements due to enhanced surface properties resulting from the increased capillary effect caused by the pores surrounded by hydrophilic fibers. The swelling test results confirmed a similar correlation between the PDA and PDA-Au groups, all of which were greater than those of PCL fibers only.

The electrical conductivity of the PDA-coated PCL fibers was enhanced by increasing the PDA coating thickness on the surface. The electrical conductivity of PCL fibers was enhanced by a hundredfold for 20PDA and a thousandfold for 20PDA-Au. The enhanced electrical conductivity resulting from PDA coating and AuNP loading provides a framework consistent with the relevant literature in areas such as tissue engineering/electroactive scaffolds and antimicrobial/sensitive surfaces, thereby presenting the material as a promising biomaterial research area.

The combination of enhanced electrical conductivity and surface hydrophilicity makes the PCL/PDA/AuNP composite fibers suitable for biomedical applications that require electroactivity. In neural and cardiac tissue engineering, the scaffold must support cell adhesion, which is facilitated by the PDA coating’s enhanced wettability. It must also allow electrical signals to circulate between cells; that is what the conductive AuNP network achieves.

## Figures and Tables

**Table 1:** Contact angle values of the electrospun fibers.

Samples	Contact Angle (°) (Mean ± Std.)
PCL	99.19 ± 4.30
0.2PDA	97.80 ± 1.82
2PDA	62.62 ± 4.75
20PDA	17.77 ± 1.21
0.2PDA-Au	28.77 ± 1.25
2PDA-Au	19.35 ± 0.07
20PDA-Au	NA

NA: Not applicable because of the sudden disappearance of the water droplet.

**Table 2:** Tensile test results of electrospun fibers.

Group	UTS (MPa) (Mean ± Std)	Fracture Strain (mm·mm^−1^) (Mean ± Std)	Elastic Modulus (MPa) (Mean ± Std)
PCL	7.66 ± 1.53	4.83 ± 0.44	43.93 ± 3.24
0.2PDA	7.57 ± 0.01	4.83 ± 0.08	44.38 ± 0.93
2PDA	8.46 ± 0.44	4.64 ± 0.11	47.12 ± 0.67
20PDA	8.84 ± 0.10	4.35 ± 0.32	48.15 ± 0.95
0.2PDA-Au	7.75 ± 0.03	5.32 ± 0.75	43.84 ± 0.72
2PDA-Au	7.87 ± 0.04	4.91 ± 0.30	46.00 ± 0.70
20PDA-Au	8.26 ± 0.43	4.21 ± 0.10	49.71 ± 0.21

**Table 3:** Surface conductivities of all groups calculated accordingly to sheet resistance values.

**Group**	**σ (S·cm^−1^)** **Mean ± Std.**
PCL	5.15 × 10^−8^ ± 2.24 × 10^−8^
0.2PDA	3.92 × 10^−7^ ± 3.04 × 10^−7^
2PDA	1.89 × 10^−7^ ± 3.20 × 10^−8^
20PDA	4.26 × 10^−6^ ± 1.94 × 10^−6^
0.2PDA-Au	1.28 × 10^−6^ ± 2.59 × 10^−7^
2PDA-Au	2.03 × 10^−5^ ± 1.76 × 10^−7^
20PDA-Au	5.11 × 10^−5^ ± 1.77 × 10^−6^

## Data Availability

The original contributions presented in this study are included in the article and Supplementary Materials. Further inquiries can be directed to the corresponding author.

## Supplementary Materials

The following supporting information can be downloaded at: https://www.mdpi.com/article/10.3390/polym17233192/s1, Table S1: One-way ANOVA test results based on the “UTS” value between groups; Table S2: One-way ANOVA test results based on the “Fracture Strain” value between groups; Table S3: One-way ANOVA test results based on the “Modulus of Elasticity” value between groups; Figure S1: Representative images of tensile test samples before and during testing; Figure S2: Representative images of contact angle measurements; Figure S3: Representative Force-Elongation plots recorded during tensile tests; Video S1: video recorded during contact angle measurement for 20PDA-Au.
