# Supplementary material for: Enhancing the Electrical Conductivity of Electrospun PCL Fibers by Coating with Polydopamine and In Situ Gold Nanoparticles Doped on the Polydopamine Coating
Source: Polymers (Basel). 2025 Nov 29;17(23):3192. doi: 10.3390/polym17233192 (PMC12693823; doi:10.3390/polym17233192)
Supplement: Supplementary file 1 [file polymers-17-03192-s001.zip › Tables S1-3.docx]

**Table S1.** One-way ANOVA test results based on the “UTS” value between groups

| **UTS (MPa)** | **Sum of Squares** | **Mean Square** | **F** | **Sig.** |
| --- | --- | --- | --- | --- |
| **Between Groups** | **4.537** | **.756** | **.980** | **.477** |
| **Within Groups** | **10.035** | **.772** |  |  |
| **Total** | **14.573** |  |  |  |

**Table S2.** One-way ANOVA test results based on the “Fracture Strain” value between groups

| **Fracture Strain** | **Sum of Squares** | **Mean Square** | **F** | **Sig.** |
| --- | --- | --- | --- | --- |
| **Between Groups** | **1.956** | **.326** | **2.400** | **.088** |
| **Within Groups** | **1.766** | **.136** |  |  |
| **Total** | **3.722** |  |  |  |

**Table S3.** One-way ANOVA test results based on the “Modulus of Elasticity” value between groups

| **Elastic Modulus (MPa)** | **Sum of Squares** | **Mean Square** | **F** | **Sig.** |
| --- | --- | --- | --- | --- |
| **Between Groups** | **85.717** | **14.286** | **3.897** | **.019** |
| **Within Groups** | **47.653** | **3.666** |  |  |
| **Total** | **133.370** |  |  |  |
