# Supplementary figures and images for: Enhancing the Electrical Conductivity of Electrospun PCL Fibers by Coating with Polydopamine and In Situ Gold Nanoparticles Doped on the Polydopamine Coating
Source: Polymers (Basel). 2025 Nov 29;17(23):3192. doi: 10.3390/polym17233192 (PMC12693823; doi:10.3390/polym17233192)

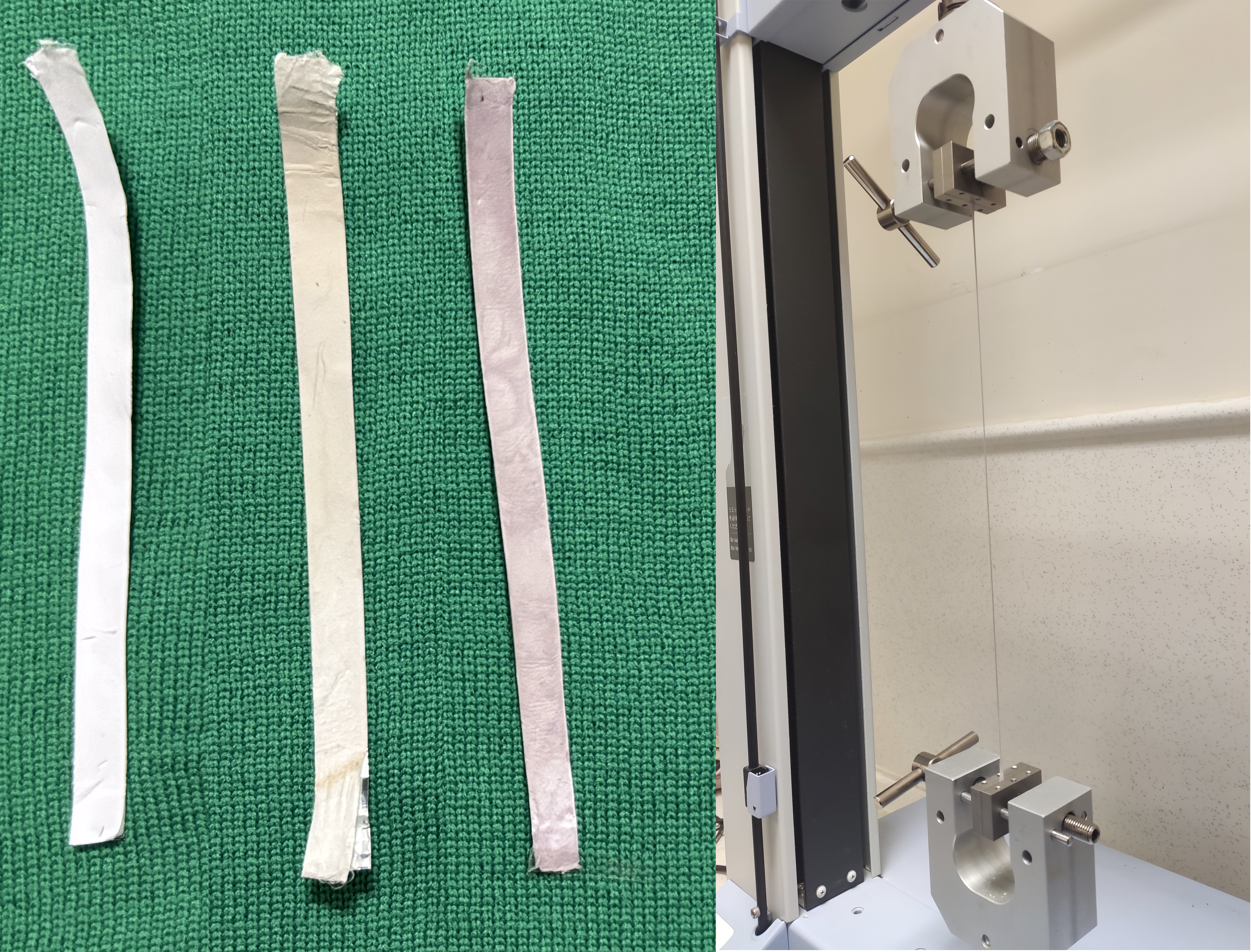

Supplement: Supplementary file 1 [file polymers-17-03192-s001.zip › FigureS1.tif]

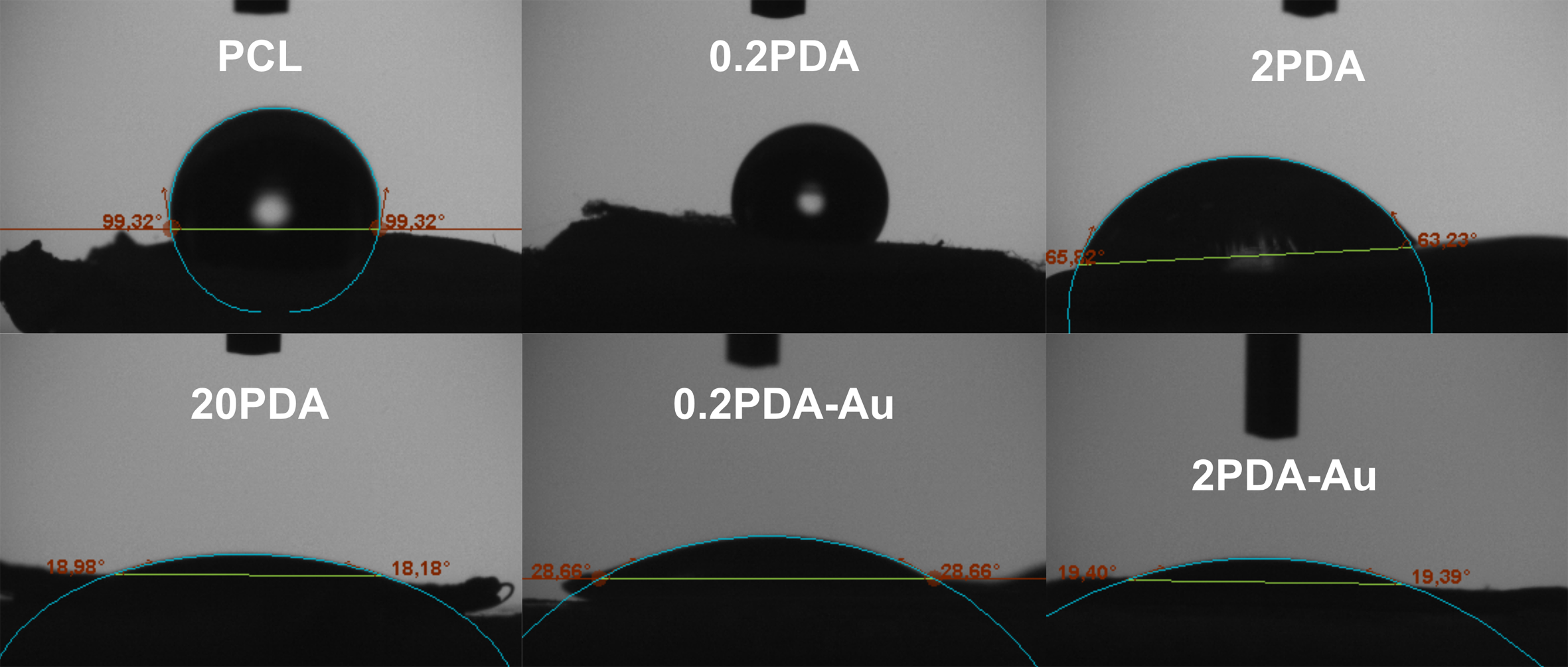

Supplement: Supplementary file 1 [file polymers-17-03192-s001.zip › FigureS2.tif]

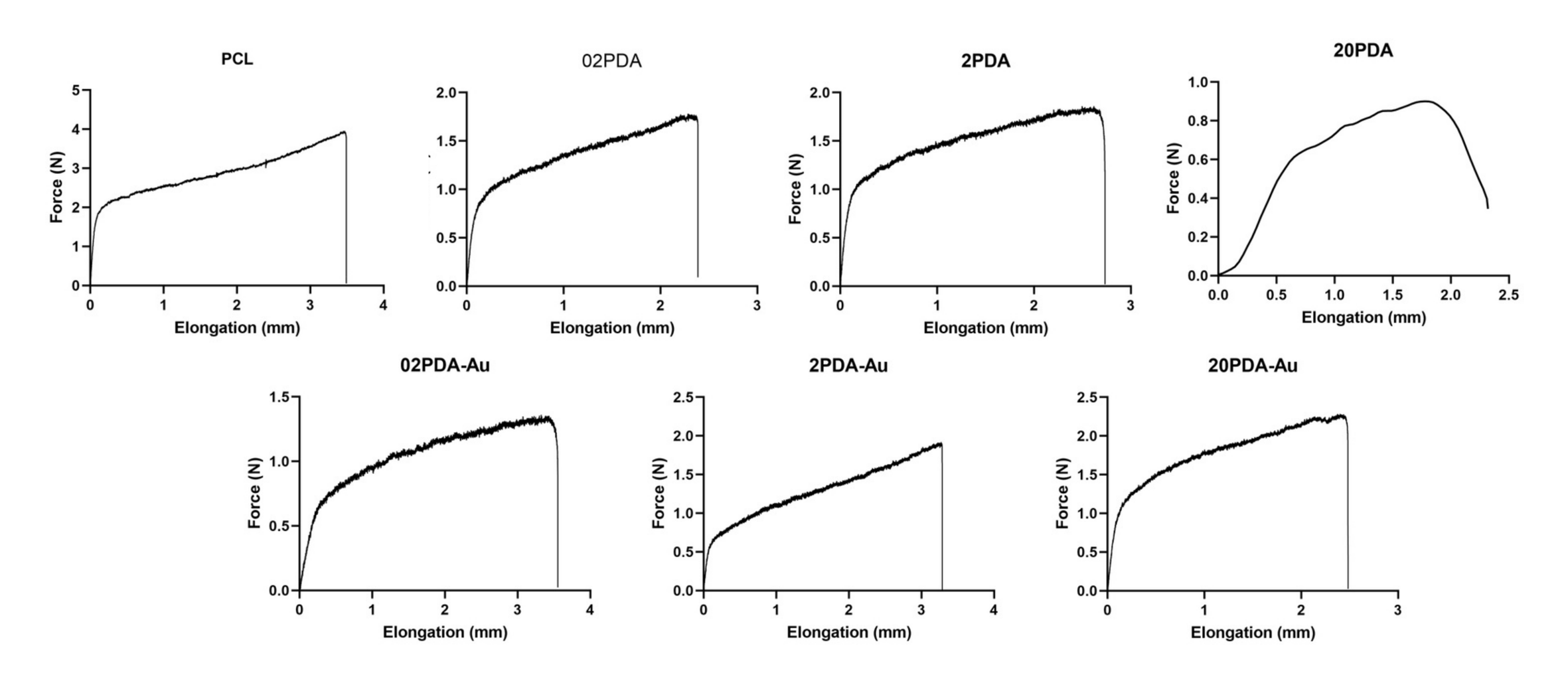

Supplement: Supplementary file 1 [file polymers-17-03192-s001.zip › FigureS3.tif]
